# Status epilepticus induced by treatment with dopamine agonist therapy for giant prolactinoma: a case report

**DOI:** 10.1186/s13256-018-1939-x

**Published:** 2019-01-20

**Authors:** Motofumi Koguchi, Yukiko Nakahara, Ryo Ebashi, Atsushi Ogata, Shoko Shimokawa, Jun Masuoka, Tatsuya Abe

**Affiliations:** Department of Neurosurgery, Faculty of Medicine, Saga University, 5-1-1 Nabeshima, Saga, 849-8501 Japan

**Keywords:** Epilepsy, Giant prolactinoma, Cabergoline, Case report

## Abstract

**Background:**

Dopamine agonists are the standard first-line medical therapy for prolactinoma. We report a rare case of giant prolactinoma with a first epileptic seizure due to rapid reduction of the tumor as a complication of dopamine agonist therapy.

**Case presentation:**

A 27-year-old Japanese man presented to our institution with a history of visual disturbance for 1 year and general fatigue for 3 months. Magnetic resonance imaging showed a tumor that arose from the pituitary and extended to the bilateral anterior skull base, the clivus, and the cavernous sinus, with compression of the optic chiasm and the bilateral frontal and temporal lobes. On the basis of the patient’s serum concentration of prolactin, we diagnosed a prolactinoma and started dopamine agonist therapy with cabergoline. The patient had a general seizure immediately after starting dopamine agonist therapy and required general anesthetic treatment following the rapid reduction of the tumor. We speculated that the rapid reduction of the tumor resulted in the retraction of the surrounding brain structure, and the epileptic seizure was then induced by dopamine agonist therapy.

**Conclusions:**

We report a rare case of giant prolactinoma with a first epileptic seizure immediately after the initiation of dopamine agonist therapy. Clinicians need to be aware that the rapid reduction of a giant prolactinoma by dopamine agonist therapy may cause an epileptic seizure.

## Background

Prolactin (PRL)-secreting pituitary adenomas (PRLomas) are the most common pituitary secreting tumors, accounting for 32–45% of all pituitary tumors [1–3]. Micro-PRLomas are more common in females, whereas macro-PRLomas are more common in males [1, 3]. Although there is no consensus on the definition of giant PRLomas, some studies have defined giant PRLoma as measuring more than 4 cm [4, 5]. In addition to pituitary hormone abnormalities, giant PRLomas cause several symptoms, such as visual field defects by compression of the optic chiasm, cranial nerve palsies by extension into the cavernous sinus, obstructive hydrocephalus, epilepsy by temporal lobe extension, and dementia by frontal lobe extension.

Dopamine agonist (DA) therapy is the standard treatment for PRLoma. In previous reports, DA therapy was the first-line medical treatment even for giant PRLomas, because normalization of PRL and significant tumor shrinkage were achieved in the majority of cases [4, 5]. The most common side effects of DA therapy are headache, nausea and vomiting, orthostatic hypotension, and depression. Attention also needs to be paid to rare complications, such as cerebrospinal fluid rhinorrhea [6, 7] and optic chiasm herniation [8–10]. However, medical treatment for PRLomas has not been known to cause seizure attacks as a side effect of DA therapy. In this article, we present a rare case of a patient with epileptic seizures immediately after the initiation of DA therapy with cabergoline (CAB). We review the previous literature and suggest the mechanism of this complication.

## Case presentation

**Fig. 1:**
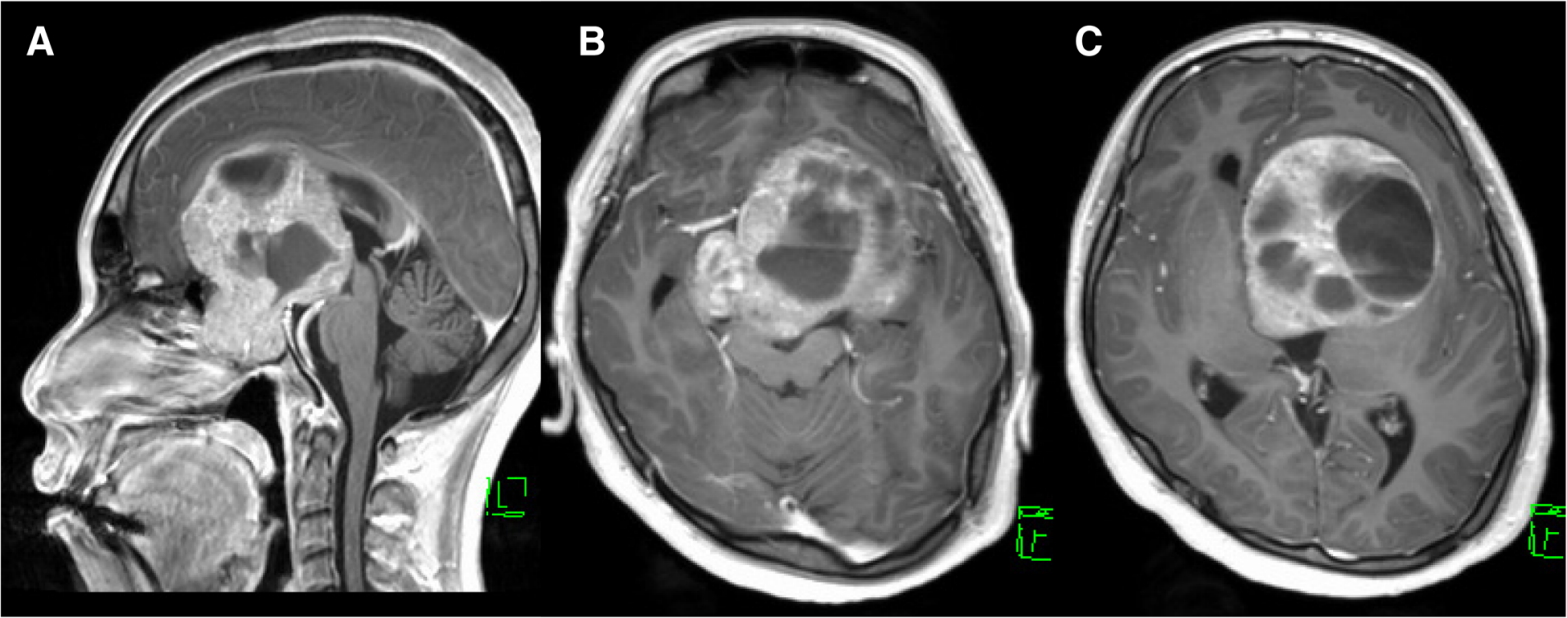
Gadolinium-enhanced T1-weighted images before the initiation of dopamine agonist therapy. **a** Sagittal view. **b** and **c** Axial images. Magnetic resonance imaging showed a tumor that arose from the pituitary and extended bilaterally through the anterior skull base, the clivus, and the cavernous sinus

A 27-year-old Japanese man with mild mental developmental retardation presented with a 1-year history of bilateral visual impairment as well as a 3-month gradually progressive general fatigue. He had no history of epileptic seizures. Neurological examination revealed blindness of the left eye, half-blindness of the right eye on the ear side, and cognitive dysfunction according to the Mini Mental State Examination 21/30. Fundus examination revealed no papilledema. Magnetic resonance imaging (MRI) revealed a 77 × 63 × 85-mm tumor that arose from the pituitary and extended bilaterally through the anterior skull base, the clivus, and the cavernous sinus, with compression of the optic chiasm and the bilateral frontal and temporal lobes (Figs. 1 and 3a–c). The patient was administered antiepileptics, such as 1000 mg/day levetiracetam, for prevention of seizure attack. His hormone profile showed hyperprolactinemia 25,270.0 ng/ml (3.6–12.8 ng/ml) and dysfunction of the other pituitary hormones (testosterone, < 0.04 ng/ml [1.3–8.7 ng/ml]; follicle-stimulating hormone, 0.54 mIU/ml [2.0–8.3 mIU/ml]; luteinizing hormone, < 0.10 mIU/ml [0.79–5.7 mIU/ml]; thyroid-stimulating hormone, 4.94 μIU/ml [0.5–5.0 μIU/ml]; free thyroxine 4, 0.6 ng/dl [0.9–1.7 ng/dl]; growth hormone, 0.22 ng/ml [0.0–2.5 ng/ml]; and adrenocorticotropic hormone, 1.7 pg/ml [7.2–63.3 pg/ml]) (Table 1). The patient received a diagnosis of a giant PRLoma with hypopituitarism. We started DA therapy with CAB 0.25 mg once per week, supplemented by daily oral hydrocortisone.

**Table 1:** Hormone profile showing hyperprolactinemia and dysfunction of the other pituitary hormones before treatment

Hormone	Value	Normal reference range
PRL	25,270.0 ng/ml	(3.6–12.8)
Testosterone	< 0.04 ng/ml	(1.3–8.7)
FSH	0.54 mIU/ml	(2.0–8.3)
LH	< 0.10 mIU/ml	(0.79–5.7)
TSH	4.94 μIU/ml	(0.5–5.0)
fT4	0.6 ng/dl	(0.9–1.7)
GH	0.22 ng/ml	(0.0–2.5)
ACTH	15.4 pg/ml	(7.2–63.3)

*Abbreviations: ACTH* Adrenocorticotropic hormone, *FSH* Follicle-stimulating hormone, *fT4* Free thyroxine, *GH* Growth hormone, *LH* Luteinizing hormone, *PRL* Prolactin

**Fig. 2:**
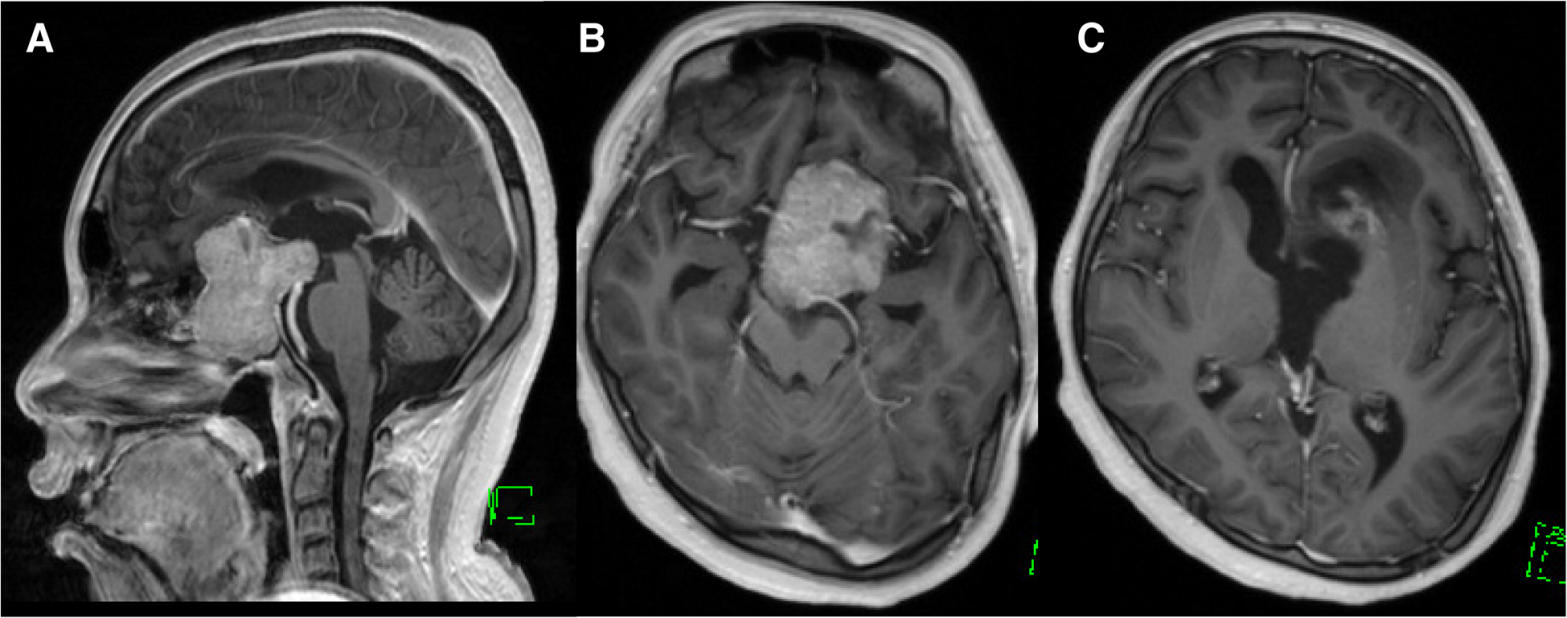
Gadolinium-enhanced T1-weighted images after dopamine agonist (DA) therapy. **a** Sagittal view. **b** and **c** Axial images. Tumor reduced significantly after the initiation of DA therapy with cabergoline

**Fig. 3:**
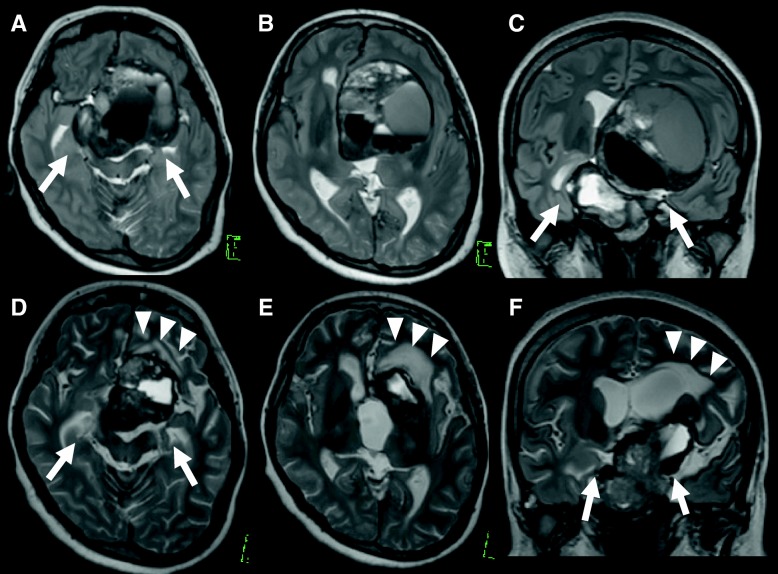
T2-weighted images (T2WI) before and after dopamine agonist therapy. **a**–**c** Before treatment. **d**–**f** After initial treatment. Following significant reduction of the tumor, the bilateral mesial temporal lobes returned to medial position (*white arrows*), and a hyperintense area on T2WI appeared in the left frontal lobe (*white arrowheads*)

Eight days after starting DA therapy, the patient had a tonic-clonic seizure with loss of consciousness that developed into status epilepticus. Incubation and general anesthetic therapy were required. The patient was admitted to the intensive care unit. An electroencephalographic examination was continuously performed; however, no findings of epileptic changes were found after general anesthesia. There was no abnormality in the laboratory analysis that may have led to status epilepticus. The patient’s blood level of PRL markedly decreased from 25,270.0 to 948.2 ng/ml. MRI revealed significant reduction of the tumor in a short period without pituitary apoplexy, including hemorrhagic or ischemic change (Figs. 2 and 3c, e, f). According to the significant reduction of the tumor, the bilateral mesial temporal lobes returned to medial position. Further, a hyperintense area in left frontal lobe appeared on T2 -weighted images (Fig. 3). Because of the possibility that the epileptic seizures were induced by the rapid shrinkage of the tumor, we suspended DA therapy until the seizures were under control with the antiepileptic drug levetiracetam 2000 mg/day. General anesthetic therapy was required for the control of seizures for 2 weeks. After 4 weeks, we resumed DA therapy with extremely low doses of CAB. Both the level of PRL and the tumor size were gradually reduced without further seizures. The patient was able to return to daily life with medication of antiepileptics and oral hydrocortisone and levothyroxine.

## Discussion

Giant PRLomas cause neurologic complications due to their massive extension into the surrounding structures. The aim of the treatment of giant PRLomas is rapid improvement of neurologic symptoms, such as visual disorders, increased intracranial pressure, and palsies of the cranial nerves. The treatment is required to reduce the size of the tumor remarkably. There are several therapeutic approaches, including DA therapy, surgery, radiotherapy, and combinations of these therapies. We chose DA therapy alone in our patient’s case for two reasons: (1) According to previous reports, DA is the first-line medical treatment even for giant PRLomas, and (2) surgical resection may cause severe complications in important surrounding structures [4, 5]. CAB has emerged as a drug for DA therapy owing to its excellent efficacy and safety. Headache, nausea and vomiting, orthostatic hypotension, depression, and cerebrospinal fluid rhinorrhea are known as common side effects of DA therapy [6, 7]. However, a small proportion of patients have rare complications, including herniation of the optic chiasm, each cerebral lobe, and the brainstem [8–10]. Moles Herbera *et al*. reported a giant PRLoma showing pons herniation into the skull base caused by tumor shrinkage after DA therapy [11]. In addition, some authors reported cases of frontal lobe herniation as a rare complication of DA therapy (Table 2) [8–10, 12].

**Table 2:** Reported cases of complication of dopamine agonist therapy for prolactinoma

Author (year) [reference]	Patient age (years)/sex	PRL (μg/L) at diagnosis	CAB dosage (mg/week)	Complication of DA therapy	
				Location of herniation	Symptoms
Papanastasiou *et al*. (2014) [8]	42/M	2000	1.5	Optic chiasma	Visual field loss
Dhanwal *et al*. (2011) [9]	36/M	183	1.0	Frontal lobe and optic chiasma	Visual field loss, seizure
Raverot *et al*. (2009) [10]	64 /M	1785	1.0	Optic chiasma	Visual field loss
	30 /M	660	3.0		
	57 /M	482	3.0		
Herbera *et al*. (2015) [11]	59 /M	1108	1.0	Pons	Dysarthria, a left hemiplegia

*Abbreviations: CAB* Cabergoline, *DA* Dopamine agonist, *PRL* Prolactin

According to the package insert of CAB, neurologic and psychiatric side effects were somnolence, aggression, and psychotic behavior. An increase in the risk of epilepsy has not been known as a side effect of CAB. Most previous reports of PRLoma described epileptic seizures as initial symptoms before treatment. Deepak *et al.* reported that epilepsy often occurred in patients with invasive macro-PRLomas and that DA therapy could reduce the frequency of seizures and the doses of antiepileptic drugs [12].

In our patient, however, epileptic seizures occurred immediately after DA therapy for giant PRLoma without a previous history of seizures. The mechanism of the epileptic seizure in our patient is speculative and might be multifocal. On the basis of his clinical course, we concluded that the rapid reduction of the tumor by DA therapy, resulting in retraction of brain matter, especially the temporal lobe around the tumor, could have been the mechanism of the epileptic seizures. This mechanism is a known cause of brain herniation with DA therapy for giant PRLomas [8, 9, 11]. The appearance of a hyperintense area in left frontal lobe on T2-weighted images may support our hypothesis of the pathogenic mechanisms of the epileptic seizure in our patient. Clinicians should be aware that DA therapy for PRLoma which is effective therapy, can induce epileptic seizures. Furthermore, medical therapies and chemotherapies with excellent efficacy for brain tumors, such as germinomas, may be a risk factor for epileptic seizure as a side effect following rapid reduction of the tumor, similarly to DA therapy for PRLoma.

## Conclusions

We report a rare case of a patient with giant PRLoma with epileptic seizures immediately after the initiation of DA therapy with CAB. It is possible that rapid tumor reduction induces epileptic seizures by the same mechanism as brain herniation. Even if there is no history of epilepsy, clinicians need to be aware of epileptic seizures during DA therapy for giant PRLomas.

## Abbreviations

ACTH: Adrenocorticotropic hormone
CAB: Cabergoline
DA: Dopamine agonist
FSH: Follicle-stimulating hormone
fT4: Free thyroxine
GH: Growth hormone
LH: Luteinizing hormone
MRI: Magnetic resonance imaging
PRL: Prolactin
PRLoma: Prolactinoma
T2WI: T2-weighted images
